# Epidemiology of Eating Disorders: Incidence, Prevalence and Mortality Rates

**DOI:** 10.1007/s11920-012-0282-y

**Published:** 2012-05-27

**Authors:** Frédérique R. E. Smink, Daphne van Hoeken, Hans W. Hoek

**Affiliations:** 1 Parnassia Bavo Psychiatric Institute, Kiwistraat 43, NL-2552 DH The Hague, The Netherlands; 2 Department of Psychiatry, University Medical Center Groningen, University of Groningen, Groningen, The Netherlands; 3 Department of Epidemiology, Mailman School of Public Health, Columbia University, New York, United States; 4 Altrecht Eating Disorders Rintveld, Altrecht Mental Health Institute, Zeist, The Netherlands

**Keywords:** Epidemiology, Incidence, Prevalence, Mortality, Eating disorders, Anorexia nervosa, Bulimia nervosa, Eating disorder not otherwise specified, Binge eating disorder

## Abstract

Eating disorders are relatively rare among the general population. This review discusses the literature on the incidence, prevalence and mortality rates of eating disorders. We searched online Medline/Pubmed, Embase and PsycINFO databases for articles published in English using several keyterms relating to eating disorders and epidemiology. Anorexia nervosa is relatively common among young women. While the overall incidence rate remained stable over the past decades, there has been an increase in the high risk-group of 15–19 year old girls. It is unclear whether this reflects earlier detection of anorexia nervosa cases or an earlier age at onset. The occurrence of bulimia nervosa might have decreased since the early nineties of the last century. All eating disorders have an elevated mortality risk; anorexia nervosa the most striking. Compared with the other eating disorders, binge eating disorder is more common among males and older individuals.

## Introduction

Epidemiological studies provide information about the occurrence of disorders and trends in the frequency of disorders over time. For epidemiological studies on eating disorders there are some methodological issues. Eating disorders are relatively rare among the general population and patients tend to deny or conceal their illness and avoid professional help. This makes community studies costly and ineffective. Therefore, many epidemiological studies use psychiatric case registers or medical records from hospitals in a circumscribed area. This type of study will underestimate the occurrence of eating disorders in the general population, because not all patients will be detected by their general practitioner or referred to the hospital or mental health care. Furthermore, differences in rates over time could be due to improved case detection, increased public awareness leading to earlier detection and wider availability of treatment services, instead of a true increase in occurrence [1, 2].

Anorexia nervosa (AN) and bulimia nervosa (BN) are the two specified eating disorders according to the *Diagnostic and Statistical Manual of Mental Disorders Fourth Edition* (DSM-IV). However, the most common eating disorder diagnosis in either clinical and community samples is the rest category ‘eating disorder not otherwise specified’ (EDNOS) [3–7]*.* EDNOS is a heterogeneous, not well defined group of eating disorders and includes partial syndromes of AN and BN, purging disorder and binge eating disorder (BED). A comprehensive meta-analysis of 125 studies suggests that EDNOS is associated with substantial psychological and physiological morbidity, comparable with the specified eating disorders [8]. In 2013 the fifth edition of the DSM is scheduled to appear, including a thoroughly revised eating disorder section. A major goal is to reduce the size of the EDNOS-category. To achieve this goal the criteria for AN and BN will be broadened [9, 10] and BED will be added as a specific eating disorder. The decision to make BED a separate diagnosis is partly informed by epidemiological data supporting the construct validity of BED. BED differs from AN and BN in terms of age at onset, gender and racial distribution, psychiatric comorbidity and association with obesity. BED is often seen in obese individuals, but is distinct from obesity per se regarding levels of psychopathology, weight- and shape concerns and quality of life [11]. BED aggregates strongly in families independently of obesity, which may reflect genetic influences [12, 13].

In this review we will describe the epidemiology of AN, BN, EDNOS and BED according to DSM-IV and – if available – to the proposed DSM-5-criteria. The proposed changes in DSM-5 diagnostic criteria will alter the coverage of the diagnostic categories and thus their disease frequencies as well. Some studies used both a narrow and a broad or partial definition of AN, including DSM-IV AN with or without amenorrhea and ICD-10 atypical AN [14–16]. These broad or partial definitions of AN are in line with the proposed DSM-5-criteria for AN and will be referred to as ‘broad AN’ throughout this review [9]. In a Finnish study of female twins, the 5-year clinical recovery rates of AN and broad AN were almost the same; i.e. 66.8 % and 69.1 % respectively, providing evidence for the validity of broad AN [14]. Definitions of each epidemiological measure are provided at the respective paragraphs.

This article is based on research publications on the epidemiology of eating disorders and updates our previous reviews, with special emphasis on studies published in the last three years [2, 17–19].

## Method

We searched online Medline/Pubmed, Embase and PsycINFO databases using several keyterms relating to eating disorders and epidemiology. The reference lists of the articles found were checked for any additional articles missed by the database search. This review is limited to articles published in English, describing the basic epidemiological parameters incidence, prevalence and mortality rates.

### Incidence

The incidence rate is the number of new cases of a disorder in the population over a specified period. The incidence rate of eating disorders is commonly expressed in terms of per 100 000 persons per year (person-years). The study of new cases provides clues to etiology.

#### Anorexia Nervosa

Community studies assessing the incidence of eating disorders are scarce. Keski-Rahkonen and colleagues conducted a large community study to quantify the incidence of AN, yielding an incidence rate of 270 per 100 000 person-years in 15–19 year old Finnish female twins during 1990–1998 [14, 19]. The incidence rate of broad AN was 490 per 100 000 person-years in the same group [14]. A much higher incidence rate of 1204 per 100 000 person-years (95 % confidence interval (CI): 652-2181) for broad AN in females aged 15–18 was found in another Finnish study of a relatively small sample of 595 adolescents [20]. The high incidence rate might be explained by the small sample size limiting statistical power and a very broad definition of AN used in this study, including subjects with an age-adjusted body mass index (BMI) up to 19, without explicitly stating that weight loss of at least 15 % had to be present. Community rates are much higher than incidence rates derived from primary care and medical records [1, 21], reflecting the selection filters that form the pathway to (psychiatric) care [22].

Incidence rates derived from general practices represent eating disorders at the earliest stage of detection by the health care system. Currin and colleagues [23] searched the General Practice Research Database in the UK for new cases of AN between 1994 and 2000 and compared their data with the findings of a similar study for 1988–1993 [24]. The age-adjusted and sex-adjusted incidence rate of AN remained stable over the two study periods: In 2000 it was 4.7 (95 % CI: 3.6-5.8) per 100 000 person-years, compared with 4.2 (95 % CI: 3.4-5.0) per 100 000 person-years in 1993. In the Netherlands the overall incidence rate of AN ascertained by general practitioners in a large representative sample of the Dutch population remained stable as well. In 1995–1999 it was 7.7 (95 % CI: 5.9-10.0) per 100 000 person-years, practically the same as the rate of 7.4 per 100 000 person-years in 1985–1989 [1]. Incidence rates are highest for females aged 15–19 years. They constitute approximately 40 % of all cases, resulting in an incidence rate of 109.2 per 100 000 15–19 year old girls per year in 1995–1999 [1]. The incidence of AN among males was less than 1 per 100 000 person-years in general practices in the Netherlands and the UK [1, 23]. AN does occur among children <13 years of age, but is relatively rare [1, 23]. Three studies used a national Paediatric Surveillance System to identify new cases of early-onset eating disorders presenting to pediatricians [25•, 26•, 27]. In Canada, the incidence rate of early-onset restrictive eating disorders diagnosed by pediatricians was 2.6 (95 % CI: 2.1-3.2) per 100 000 person-years in children aged 5 to 12 years [25•]; in Australia it was 1.4 (95 % CI: 1.1-1.7) per 100 000 person-years in 5–13 year old children [27]. In the Canadian study 62 % of new restrictive eating disorder cases met criteria for AN [25•]. Of the Australian pediatric inpatients with a newly diagnosed restrictive eating disorder only 37 % could be classified as AN, although 61 % had life-threatening complications of malnutrition [27]. In British pediatric and psychiatric care an overall incidence rate of 1.1 per 100 000 person-years for AN was found among children <13 years of age [26•]. Among middle aged and elderly women AN is relatively rare as well [28–30]. In a Spanish population-based study using the Public Health Registry to identify eating disorder cases diagnosed by mental health professionals, new cases of AN were found among women over 45 years of age, constituting 64 % of all new eating disorder diagnoses in this age-group [31]. It is unknown whether this reflects late detection or late age at onset.

The question of whether the incidence of AN is on the rise has been under debate. Longterm epidemiological studies are sensitive to minor changes in the absolute incidence numbers and in the methods used, for example, variations in registration policy, demographic differences between the populations, faulty inclusion of readmissions, the specific methods of detection used or the availability of services [18, 32].

**Fig. 1:**
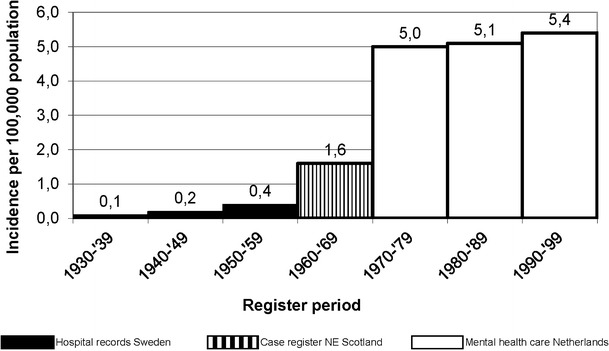
Registered Yearly Incidence of Anorexia Nervosa. *Adapted from* Hoek [18]

In a meta-analysis of the incidence of AN in mental health care, various studies in northern Europe were combined (see figure 1).

Until the 1970s, there was an increase of the registered incidence of AN in Europe. Since 1970, the incidence of AN in Europe seems to have been rather stable [1, 18, 33, 34]. In Switzerland the incidence of severe AN in females was studied in a geographically defined region using the same methodology from 1956 to 1995. The incidence of severe AN requiring hospital admission rose significantly during the 1960s and 1970s and reached a plateau of around 1.2 per 100 000 person-years thereafter [21].

In the Netherlands from the 1980s up to now general practitioners have registered new cases with an eating disorder in a representative sample of the Dutch population. While the overall incidence of AN was stable around 7 per 100 000 person-years, the incidence in 15–19 year old girls increased significantly from 56.4 per 100 000 person-years in 1985–1989 to 109.2 per 100 000 person-years in 1995–1999 [1]. This is in line with an Italian study examining age at onset of AN in a large sample of 1,666 patients referred to an eating disorders outpatient unit between 1985 and 2008. Patients referred in more recent years had an earlier age at onset [35•]. In Rochester, MN, USA, the age-adjusted incidence rates of AN showed a significant linear increasing trend only in females aged 15–24 years from 1935–1989 [36].

#### Bulimia Nervosa

Only a few incidence studies of BN have been conducted. In the community study of the 1975–1979 birth cohorts of female Finnish twins the incidence rate of BN was 200 per 100 000 person-years at the peak age of incidence, 16–20 years [37••]. A broader definition of BN was examined as well. When symptom frequency was relaxed to once a week, in concordance with the proposed DSM-5-criteria [10], the peak incidence rate of broad BN was 300 per 100 000 person-years in 16–20 year old females [37••]. Isomaa and colleagues found an incidence rate of 438 (95 % CI: 132–1175) per 100 000 person-years in 15–18 year old Finnish females for another broad definition of BN, including subjects who fulfilled all but one of the criteria for BN [20] .

According to the nation-wide primary care study in the Netherlands, the overall incidence rate of BN tended to decrease from 8.6 per 100 000 person-years in 1985–1989 to 6.1 per 100 000 person-years in 1995–1999 [1]. In a primary-care study from the UK, the overall age- and sex-adjusted incidence rate of BN decreased during the second half of the 1990s from 12.2 per 100 000 person-years in 1993 to 6.6 per 100 000 person-years in 2000. However, the incidence rate of BN in women aged 10–19 years remained relatively stable around 40 per 100 000 person-years in 1993 as well as in 2000 [23].

Several studies suggest that age at onset of BN is decreasing. In a sample of 793 Italian BN patients referred to an eating disorders outpatient unit between 1985 and 2008, subjects born in 1970–1972 had a mean age at onset of 18.5 years, compared to 17.1 years in subjects born between 1979–1981 [35•]. In the Dutch primary care study the high risk group of BN shifted from 25–29 year old females in 1985–1989 to 15–24 year old females in 1995–1999 [1]. It is unclear whether this reflects a true earlier age at onset or rather earlier detection of BN cases.

#### Eating Disorder Not Otherwise Specified and Binge Eating Disorder

Epidemiological studies on EDNOS are sparse, because of its heterogeneity and undefined operational criteria, except for BED, for which in DSM-IV research criteria were formulated. In a Spanish population-based study using the Public Health Registry to identify eating disorder cases diagnosed by mental health professionals, the incidence rate of EDNOS was 6.5 (95 % CI: 4.8-7.9) per 100 000 inhabitants per year [31]. A British national surveillance study of newly diagnosed eating disorders in pediatric and psychiatric care found an incidence rate of 1.2 per 100 000 person-years for EDNOS among children <13 years [26•]. To our knowledge no incidence studies on BED yet exist. Binge eating as a disordered eating behavior or symptom is quite common among adolescents: In a longitudinal study of a large cohort of US adolescents, the incidence rate for binge eating was 10.1 per 1000 person-years among females and 6.6 per 1000 person-years among males (both sexes ≥ 14 years), which translates into 1010 per 100 000 person-years and 660 per 100 000 person-years among female and male adolescents, respectively [38].

### Prevalence

The prevalence can be expressed as point prevalence, one-year prevalence rate and lifetime prevalence. The point prevalence is the prevalence at a specific point in time, e.g. January 1 of a specific year. The one-year prevalence rate is the point prevalence plus annual incidence rate (the number of new cases in the following year). The lifetime prevalence is the proportion of people that had the disorder at any point in their life. The prevalence is the most useful measure for planning health care facilities, as it indicates the demand for care. Case detection through a two-stage screening approach is the standard procedure to estimate the prevalence of eating disorders [2, 39]. In the first stage a large population is screened for the likelihood of an eating disorder by means of a screening questionnaire that identifies an at-risk group. In the second stage definite cases in the at-risk group are established on the basis of a personal interview. Problems associated with this type of study are poor response rates, sensitivity of the screening instrument and the restricted size of the groups interviewed [40]. To circumvent this last problem several studies use a structured interview such as the Composite International Diagnostic Interview (CIDI), usually administered by lay-interviewers, to assess the prevalence of eating disorders in a large population sample.

#### Anorexia Nervosa

The lifetime prevalence of AN and broad AN has been assessed in three large population-based cohort studies of twins [14–16]. In Sweden, it was 1.2 % (AN) and 2.4 % (broad AN) in the largest twin study of women from the 1935–1958 birth cohorts [16]. In an Australian study of female twins aged 28–39 years, the lifetime prevalence of AN was 1.9 % and of broad AN 4.3 % [15]. The lifetime prevalence of AN was 2.2 % and of broad AN 4.2 % in a large sample of women from the 1975–1979 birth cohorts of Finnish twins [14]. In men from the same birth cohort the lifetime prevalence of AN was 0.24 % [41].

Stice and colleagues followed a relatively small sample of 496 adolescent girls over an 8-year period from early adolescence into young adulthood, administering annual diagnostic interviews. They found a lifetime prevalence by age 20 years of 0.6 % for AN and 2.0 % for broad AN [42]. In Portugal, the point prevalence of AN among adolescent girls was 0.39 % and of broad AN 0.64 % [6]. In an Australian population-based sample of 1,597 14-year old boys and girls, only one case of AN was identified by means of a self-report eating disorder screening questionnaire; four other subjects met partial criteria for AN [43].

Prevalences of AN estimated with two-stage procedures varied from 0 to 0.9 % with an average point prevalence of 0.29 % in young females [2]. In a meta-analysis [2], the one-year prevalence rate per 100 000 young females was computed at different levels of care (Table 1).

**Table 1:** Estimates of one-year prevalence rates per 100 000 young females at different levels of care

Level of morbidity	Anorexia nervosa	Bulimia nervosa^a^
Community	370	1000
Primary care	160	150
Mental health care	127	75

^a^Based on a meta-analysis of Hoek and van Hoeken in 2003 and corrected for more recent findings of a decrease in point prevalence [44] and incidence [1] of BN

Using two-stage studies of community samples and estimates of the incidence, the one-year prevalence rate of AN in the community was calculated as 370 per 100 000 young females. One can conclude from table 1 that the majority of patients with AN in the community do not enter the mental healthcare system [18].

Several studies used the CIDI to estimate the lifetime prevalence of AN in large population samples [45, 46••, 47••]. Both in a nationally representative survey of the US household population [45] and in a population-based study in six European countries [46••], the lifetime prevalence of AN was 0.9 % among adult females. In the US study it was 0.3 % among males [45], while in the European study not a single male case of AN was found [46••]. In a large representative sample of US adolescents the lifetime prevalence of AN was 0.3 % in 13–18 year old females as well as males [47••]. The female-to-male ratio in these studies is considerably lower than the 10:1 ratio found in the Finnish twin study and as reported in previous reviews [2, 14, 41], which could be due to differences in methodology and small numbers of cases with eating disorders [45, 47••]. However, despite this restriction many recent community-based studies have found that AN is more common among males than previously thought. AN may be even more frequently underdetected in males than in females [19].

A large study of Swedish twins born during the period 1935–1958 documented a higher prevalence of AN in both male and female participants born after 1945 than those born before 1945 [16].

#### Bulimia Nervosa

The generally accepted point prevalence of BN from two-stage studies is about 1 % among young females [2, 40]. Keski-Rahkonen and colleagues found a lifetime prevalence of 1.7 % for BN in women from the 1975–1979 birth cohorts of Finnish twins [37••]. When symptom frequency was relaxed to once a week, in concordance with the proposed DSM-5-criteria, the lifetime prevalence rose to 2.3 % in women [37••]. In an Australian twin cohort of women aged 28–39 years, a lifetime prevalence of BN of 2.9 % was found [15]. According to US [45] and European [46••] large scale two-stage studies of the population the lifetime prevalence of BN, assessed with the CIDI, varied between 0.9 and 1.5 % among women and between 0.1 % and 0.5 % among men. Marques and colleagues compared the prevalence of BN across nationally representative samples of ethnic groups in the US. BN was more prevalent among Latinos and African Americans than non-Latino whites. Lifetime prevalences ranged from 0.51 % (non-Latino whites ) to 2.0 % (Latinos) [48]. In a recent study of a nationally representative sample of US adolescents, a lifetime prevalence of BN of 1.3 % and 0.5 % was found among 13–18 year old females and males, respectively [47••]. In a US sample of 496 adolescent females, followed for 8 years, a lifetime prevalence of 1.6 % for BN was found at age 20 years [42]. An Australian population-based study of 1,597 14-year old adolescents reported 9 cases of BN, translating into a point prevalence of 0.6 % [43].

Trace and colleagues assessed the impact of reducing the binge eating frequency on the lifetime prevalence of BN in a large population sample of female Swedish twins. The lifetime prevalence of BN increased from 1.2 % for a minimum of 8 binges per month (DSM-IV) to 1.6 % for at least 4 binges per month (proposal DSM-5) [49].

The decrease in occurrence of BN over time found in the incidence studies is supported by a US study of university students in which the point prevalence of BN among women significantly decreased from 4.2 % in 1982, to 1.3 % in 1992 and 1.7 % in 2002 [44]. In another US study among female students the point-prevalence of probable cases of BN remained relatively stable between 1990 and 2004 [50].

#### Eating Disorder Not Otherwise Specified and Binge Eating Disorder

Often used diagnostic interviews to estimate the prevalence of eating disorders, like the CIDI and the Structured Clinical Interview for DSM disorders (SCID) do not cover EDNOS. In recent studies that used the CIDI alterations have been made to include subthreshold AN [47••] and BED [ 45, 46••, 47••]. Researchers have operationalized EDNOS in different ways; reported prevalences are therefore difficult to compare and in community studies the use of limited definitions will underestimate the true prevalence of eating pathology that could be classified as EDNOS [8]. The point prevalence of EDNOS in a nation-wide community sample of young females was 2.4 % [6].

The lifetime prevalence of BED has been assessed in large population samples in the US [45, 47••] and Europe [46••]. In six European countries it was 1.9 % for women and 0.3 % for men [46••]. In the US higher lifetime prevalences were found among adults (women 3.5 %; men 2.0 %) and among 13–18 year old adolescents (girls 2.3 %; boys 0.8 %) [45, 47••]. The US researchers used a duration criterium of only three months instead of the six months DSM-IV research criteria require, which might partly explain the higher percentages. Hudson and colleagues examined data from a non-clinical sample to estimate how much the prevalence of BED will increase under the proposed DSM-5-criteria that relax the requiremens for the frequency (from two per week to one per week) and duration of binges (from six to three months). They extrapolated their findings to the results of the aforementioned study of the US household population and estimated that the lifetime prevalence of BED would increase with an additional 0.1 % to 3.6 % in women and 2.1 % in men [51]. In a study of a large sample of adult Swedish female twins, a relatively low lifetime prevalence of 0.17 % for BED was found, which rose to 0.35 % when DSM-5 criteria were applied [49].

### Mortality

One could describe the mortality rate as an incidence rate in which the event being measured is death [52]. Mortality rates are often used as one of the indicators of illness severity. The standard measures for mortality are the crude mortality rate (CMR) and the standardized mortality ratio (SMR). The CMR is the number of deaths within the study population over a specified period. The SMR is the ratio of observed deaths in the study population to expected deaths in the population of origin [18, 19, 52].

#### Anorexia Nervosa

In a meta-analysis of excess mortality in the 1990s, anorexia nervosa was associated with the highest rate of mortality among all mental disorders [53]. In a recent meta-analysis of 35 published studies [54••], the weighted CMR for AN was 5.1 deaths (95 % CI: 3.99-6.14) per 1000 person-years, translating into 5.1 % per decade or 0.51 % per year. One in five individuals with AN who died had committed suicide [54••]. The overall SMR was 5.86 (95 % CI: 4.17-8.26) with a mean follow-up period of 14 years. The duration of follow-up is inversely correlated with the reported SMR; as duration of follow-up increases, the expected mortality in the population of origin will increase as well, resulting in lower SMRs. In a meta-analysis of SMRs in 2001, the overall SMR of AN in studies with 6–12 years of follow-up was 9.6 (95 % CI: 7.8-11.5) and in studies with 20–40 years of follow-up 3.7 (95 % CI: 2.8-4.7) [55]. Age, case severity and study period influence mortality rates as well [54••]. In a Swedish study [56], a significantly higher mortality rate (4.4 % vs. 1.2 %) was found among female patients hospitalized due to AN in 1977–1981 compared with those hospitalized in 1987–1991. The authors argue that this dramatic decrease in mortality is related to the introduction of specialized care units for patients with eating disorders. Finally, in an audit conducted in the UK, death certificates emerged as a flawed source of information with both over- and underreporting of AN as a cause of death, the latter probably more common [19, 57].

#### Bulimia Nervosa

In a recent meta-analysis of 12 studies describing the mortality rates of patients with BN, a weighted mortality rate of 1.74 per 1000 person-years was found (95 % CI: 1.09-2.44), which means that per year 0.17 % of BN-patients die. The overall SMR was 1.93 (95 % CI: 1.44-2.59) [54••]. Crow and colleagues examined mortality in a sample of 1885 patients evaluated for treatment for eating disorders at an out-patient clinic between 1979 and 1997. Of the 906 BN-patients, 35 (3.9 %) had died after a mean follow-up of almost 19 years. Suicide accounted for 23 % of deaths [58].

#### Eating Disorder Not Otherwise Specified and Binge Eating Disorder

A recent meta-analysis of six studies describing the mortality rate of patients with EDNOS reported a weighted annual mortality rate of 3.31 deaths (95 % CI: 1.48-5.75) per 1000 person-years. The overall SMR was 1.92 (95 % CI: 1.46-2.52) [54••]. Any elevated mortality risk of EDNOS could be partly explained by the assertion that EDNOS sometimes reflects the earlier stages of AN [54••, 59]. None of the six studies stated that BED was part of the EDNOS category, which may be explained by the fact that the study periods of these studies were (in part) before the publication of DSM-IV in 1994, in which BED was introduced [58–63].

Keel and Brown reviewed six studies describing course and outcome of BED [64]. Duration of follow-up ranged from one (four studies) to 12 years (one study). The single 12-year follow-up study provided the only report of deaths at follow-up: 2 of 68 patients admitted for inpatient treatment of BED had died after 12 years, leading to a CMR of 2.9 % and a non-significant SMR of 2.29 (95 % CI: 0.00-5.45) [65]. These data from an inpatient sample may not be representative of patients with BED seen in other settings [64]. BED is associated with obesity. In a large US population-based study 42 % of the subjects with a lifetime diagnosis of BED were obese (BMI >30 kg/m^2^) at the time of the interview and also a significantly higher prevalence of morbid obesity (BMI > 40 kg/m^2^) was found among these subjects compared to respondents without any eating disorder (OR 4.9; 95 % CI: 2.2-11.0) [45]. Obesity and especially morbid obesity is associated with increased risk for mortality, although the net effect of obesity on mortality is difficult to quantify [66, 67]. Finally, in a meta-analysis of the risk of suicide in eating disorders, no suicide had occurred among 246 patients with BED after a mean follow-up of 5.3 years [68].

### Eating Disorders in Non-Western Countries and Among Ethnic Minorities

In the past, eating disorders have been characterized as culture-bound syndromes, specific to Caucasian subjects in Western, industrialized societies [69]. Recent studies demonstrate that eating disorders and abnormal eating behaviors do occur in non-Western countries and among ethnic minorities [48, 70–74]. An increasing occurrence of eating disorders in non-Western countries has been associated with cultural transition and globalization, including modernization, urbanization and media-exposure promoting the Western beauty-ideal [70, 75–77]. The most comprehensive attempt to quantify eating disorders in a non-Western setting was conducted on the Caribbean island Curaçao (Netherlands Antilles), where the full spectrum of community health and service providers was contacted. The overall incidence of AN of 1.82 (95 % CI: 0.74-2.89) per 100 000 person-years was much lower than in the US and Western Europe. No cases were found among the black population. However, the incidence of 9.08 (95 % CI: 3.71–14.45) among the minority mixed and white population was similar to the incidence in the Netherlands and in the United States [78]. In the Netherlands, incidence rates of psychiatric hospital admissions for AN did not differ between Netherlands Antilles immigrants and native Dutch [79], suggesting that exposure to the Western beauty ideal is a risk factor for the development of AN, possibly in interaction with migration-related stress. A similar finding for risk of BED among Mexican-American immigrants was found by Swanson and colleagues: In their study of a sample of people of Mexican origin in Mexico and the US, migration from Mexico to the US was associated with an increased risk of BED [80]. A recent study comparing prevalences of eating disorders across ethnic groups in the United States reported similar prevalences of AN and BED among non-Latino whites, Latinos, Asians and African Americans. BN was more prevalent among Latinos and African Americans than among non-Latino whites [48].

## Conclusions

AN is relatively common among young women. While the overall incidence rate remained stable over the past decades, there has been an increase in the high risk-group of 15–19 year old girls. It is unclear whether this reflects earlier detection of AN cases or an earlier age at onset. The occurrence of BN might have decreased since the early nineties of the last century. All eating disorders have an elevated mortality risk; AN the most striking. Compared with the other eating disorders, BED is more common among males and older individuals.
